# Targeting the T-cell membrane type-1 matrix metalloproteinase-CD44 axis in a transferred type 1 diabetes model in NOD mice

**DOI:** 10.3892/etm.2012.821

**Published:** 2012-11-20

**Authors:** ALEXEI Y. SAVINOV, ALEX Y. STRONGIN

**Affiliations:** 1 Sanford Research, University of South Dakota, Sioux Falls, SD 57105;; 2 Sanford-Burnham Medical Research Institute, La Jolla, CA 92037, USA

**Keywords:** CD44, proteolysis, type 1 diabetes, metalloproteinases, migration, T cell homing

## Abstract

This study tested the hypothesis that membrane-tethered type-1 matrix metalloproteinase (MT1-MMP)-induced proteolysis of T cell CD44 is important for defining the migration and function of autoreactive T cells, including diabetogenic, insulin-specific and K^d^-restricted IS-CD8^+^ cells. To confirm the importance of MT1-MMP proteolysis of CD44 in type 1 diabetes (T1D), the anti-diabetic effects of three MMP inhibitors (3(S)-2,2-dimethyl-4[4-pyridin-4-yloxy-benzenesulfonyl]-thiomorpholine-3-carboxylic acid hydroxamate [AG3340], 2-(4-phenoxyphenylsulfonylmethyl) thiirane [SB-3CT] and epigallocatechin-3-gallate [EGCG]) were compared using an adoptive diabetes transfer model in non-obese diabetic (NOD) mice. Only AG3340 was capable of inhibiting both the activity of MT1-MMP and the shedding of CD44 in T cells; and the transendothelial migration and homing of IS-CD8^+^ T cells into the pancreatic islets. SB-3CT and EGCG were incapable of inhibiting T cell MT1-MMP efficiently. As a result, AG3340 alone, but not SB-3CT or EGCG, delayed the onset of transferred diabetes in NOD mice. In summary, the results of the present study emphasize that the MT1-MMP-CD44 axis has a unique involvement in T1D development. Accordingly, we suggest that a potent small-molecule MT1-MMP antagonist is required for the design of novel therapies for T1D.

## Introduction

The pathogenesis of type 1 diabetes (T1D) involves the activation of autoimmune T killer cells within the pancreas-draining lymph nodes. Activated autoimmune T cells then leave the regional lymphatics, enter the bloodstream and gradually transmigrate from the bloodstream through the pancreatic endothelium and into the islets of Langerhans where they destroy insulin-producing β cells (1). The dynamic interaction of T cell CD44 with its endothelial ligand (a non-sulfated linear hyaluronan glycosaminoglycan) is essential for accomplishing the firm adhesion of T cells to the pancreatic endothelium and then for the transendothelial migration and subsequent homing of the adherent T cells into the islets (2–4).

Our previous work and the studies of others have suggested that the invasion-promoting membrane type-1 matrix metalloproteinase (MT1-MMP) (5) dynamically regulates the functionality of the cell surface-associated signaling and adhesion receptor CD44 in cancer cells and diabetogenic T cells (6–9). By means of the regulatory proteolysis of CD44, MT1-MMP mediates the transition from T cell adhesion to endothelial cells to T cell transmigration. When combined, these cellular processes result in the sustained homing of autoreactive T cells into the pancreatic islets. As a result, the efficiency of T cell homing in the islets is directly proportional to the severity of the diabetic disease. The inhibition of MT1-MMP proteolysis of CD44 drastically reduced the diabetogenic efficiency of T cells, immobilized T cells on the endothelium, repressed the homing of diabetogenic T cells into the pancreatic islets, reduced insulitis and mononuclear cell infiltration and promoted the recovery of the insulin-producing β cells in non-obese diabetic (NOD) mice with freshly developed T1D. The importance of the MT1-MMP-CD44 axis in T1D has thus been identified in a diabetes transfer model with NOD mice and in freshly diabetic NOD mice (Savinov, 2005 #78) (9).

A highly potent MMP inhibitor, 3(S)-2,2-dimethyl-4[4-pyridin-4-yloxy-benzenesulfonyl]-thiomorpholine-3-carboxylic acid hydroxamate (AG3340), has been used previously to efficiently control T cell MT1-MMP activity (6,9). The K_i_ values of AG3340 against MMP-2, MMP-3, MMP-13 and MT1-MMP are ∼100, 300, 40 and 200 pM, respectively. Other individual MMPs are significantly less sensitive to AG3340 inhibition (e.g. the K_i_ values for MMP-1 and MMP-7 are 10 and 55 nM, respectively). AG3340 was used as an oral anti-angiogenic drug in phase I–III clinical trials in humans with advanced non-small cell lung cancer and prostate cancer. The trials were halted due to the drug’s lack of effectiveness in patients with the late-stage disease (10).

To shed additional light on the physiological significance of the MT1-MMP-CD44 axis in the homing of diabetogenic T cells and also on the importance of the specific T cell MT1-MMP-dependent targeting of CD44, the anti-diabetic potencies of two broad-range non-hydroxamate MMP inhibitors [2-(4-phenoxyphenylsulfonylmethyl)thiirane (SB-3CT) and epigallocatechin-3-gallate (EGCG)] were tested using a transferred diabetes model in NOD mice. SB-3CT and EGCG, however, do not inhibit MT1-MMP efficiently. SB-3CT exhibits a dithiolate moiety that chelates the active-site zinc. While SB-3CT is an effective and selective MMP-2/MMP-9 gelatinase inhibitor, it either does not inhibit or poorly inhibits other MMPs and the closely related metalloprotease TACE (tumor necrosis factor α-converting enzyme) (11,12). EGCG, a major catechin of green tea, also exhibits inhibitory, albeit largely non-specific, effects on MMPs (13–18). Due to their proven ability to transfer diabetes to NOD mice effectively and rapidly (6,19,20), highly diabetogenic, insulin-specific, CD8-positive, K^d^-restricted T cells of the TGNFC8 clone (IS-CD8^+^ T cells) were used in the present study. The results demonstrated that the MT1-MMP-targeting inhibitor AG3340, but not SBC3T and EGCG (despite their potency against MMPs distinct from MT1-MMP), exhibited a significant anti-diabetic action. The specific effect of AG3340 demonstrates the importance of the MT1-MMP-CD44 axis in diabetogenesis, thus making T cell MT1-MMP a promising drug design target for T1D therapy.

## Materials and methods

### General reagents

Reagents were from Sigma (St. Louis, MO, USA) unless indicated otherwise. AG3340 was a gift of Dr Peter Baciu (Allergan, Irvine, CA, USA). SB-3CT (an inhibitor of MMP-2 and MMP-9) and α1-antitrypsin were purchased from Calbiochem (La Jolla, CA, USA).

### Mice and cells

NOD/LtJ mice were from the Jackson Laboratory (Bar Harbor, ME, USA). IS-CD8^+^ cells (insulin-specific, CD8-positive, K^d^-restricted T cells of the TGNFC8 clone from the NOD mouse pancreas) (20) were maintained in Click’s medium supplemented with 5% fetal calf serum, 2×10^5^ M β-mercaptoethanol, 20 mM penicillin-streptomycin, 3 mg/ml L-glutamine and 5 U/ml murine interleukin-2. Every 3 weeks, the IS-CD8^+^ cells were stimulated with irradiated NOD splenocytes (2000 Rad) loaded with the L15YLVCGERG23 insulin B chain peptide (10 μg/ml) (19).

### Induction of diabetes

Mice received AG3340 (1 mg/kg), SB-3CT or EGCG (10 or 100 mg/kg) or PBS IP. After 30 min, IS-CD8^+^ cells (1×10^7^) in PBS were injected IV into the irradiated (725 Rad, 24 h in advance) 5–8-week-old male recipient mice (5–6 animals/group). Afterwards, the mice received one injection of their respective inhibitor every other day until they developed diabetes (1–2 weeks). The onset of diabetes was monitored by measuring urine and blood glucose levels with Diastix strips and a glucose meter (Fisher Scientific, Hampton, NH, USA), respectively. Mice with urine glucose levels ≥300 mg/dl for 3 consecutive days were considered to be diabetic. The animal treatment protocols were approved by the institutional Animal Care Committee.

### Fluorescent labeling of IS-CD8^+^ cells

IS-CD8^+^ cells (1×10^7^/ml) were labeled with a fluorescent didodecyl-tetramethylindocarbocyanine perchlorate (DiI) dye. DiI-labeled cells (1×10^7^) were injected IV into irradiated (725 Rad, 24 h in advance) 5–8-week-old mice (4–5 mice/group). The mice received AG3340 (1 mg/kg), SB-3CT or EGCG (10 or 100 mg/kg) or PBS IP 30 min prior to the cell injection. After 24 h, the pancreata were removed from euthanized mice, fixed in 0.1 M periodate-lysine-paraformaldehyde phosphate buffer, sucrose-saturated and freeze-molded in OCT compound (Sakura Finetek, Torrance, CA, USA). Each pancreas was cryosectioned into 7 μm-sections separated by a 60 μm-interval. DiI-labeled cells were counted by a blinded observer using a fluorescence microscope and the cell positions relative to the islet boundary were recorded. The DiI-cells localized within the islet boundary were considered to be ‘inside’. The DiI-cells adjacent to an islet but outside of the islet boundary were considered to be ‘at the entrance’ of the islet (9,19).

### Cell biotinylation

IS-CD8^+^ cells were surface biotinylated with sulfo-NHS-LC-biotin (Pierce, Rockford, IL, USA) (9), re-suspended in serum-free Click’s medium supplemented with AG3340 (50 μM), SB-3CT (100 μM) or EGCG (50, 100 and 500 μM) and allowed to adhere for 4 h to plastic coated with 2% type I collagen. The cells were then lysed using 50 mM N-octyl-β-D-glucopyranoside (9). Biotin-labeled CD44 was captured on streptavidine-agarose beads from both the cell lysate and medium samples. The captured samples were examined by western blotting with the CD44 antibody (clone IM7.8.1; BD Biosciences, Franklin Lakes, NJ, USA).

### MMP-2 activation

IS-CD8^+^ cells (1×10^6^) in serum-free Click’s medium were supplemented with purified MMP-2 (20 ng) and allowed to either adhere for 18 h to plastic coated with 2% type I collagen or remain in solution. AG3340 (50 μM), SB-3CT (100 μM) or EGCG (50, 100 and 500 μM) were added to the cells. After 18 h, 30 μl samples of medium were withdrawn and analyzed by gelatin zymography to identify the MMP-2 status.

### Cleavage of α1-antitrypsin

α1-antitrypsin (250 ng) was co-incubated for 2 h at 37°C with p-aminophenylmercuric acetate-activated MMP-2 (7 ng) (21,22). The reactions were stopped using 2% SDS and analyzed using 10% gel electrophoresis followed by Coomassie staining.

## Results and discussion

### MT1-MMP sheds CD44 in T cells

**Figure 1.**
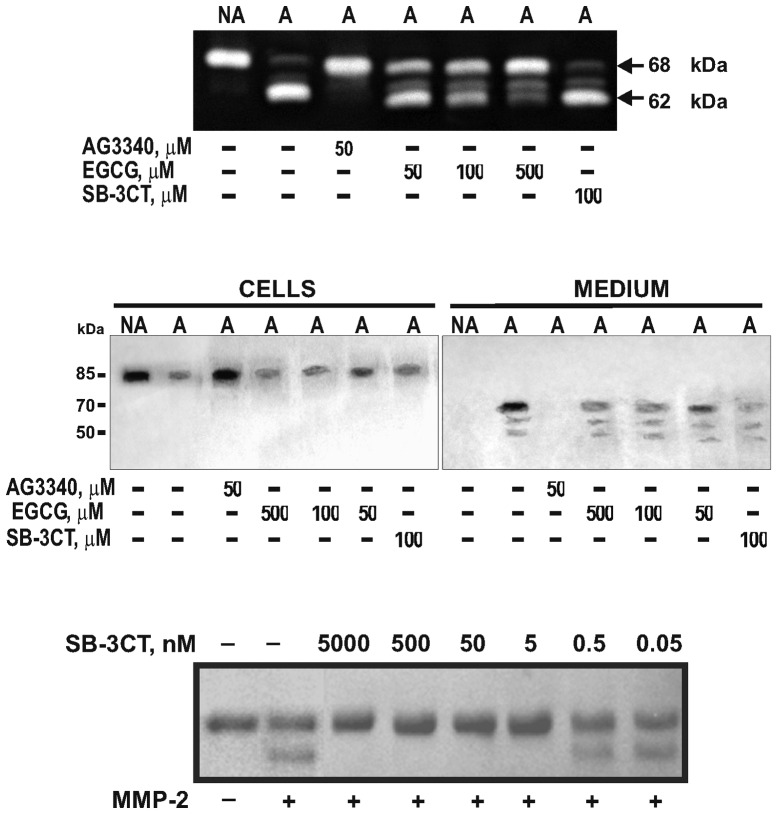
AG3340 inhibits MT1-MMP and the shedding of CD44 in IS-CD8^+^ T cells. (Upper panel) Gelatin zymography of MMP-2. To analyze the activation of MMP-2 by cellular MT1-MMP, adherent (A) and non-adherent (NA) IS-CD8^+^ cells were each incubated for 18 h in serum-free medium. Purified MMP-2 (20 ng) was added to the cells. The activation of MMP-2 was analyzed by gelatin zymography of the medium aliquots to observe the conversion of the 68 kDa MMP-2 proenzyme into the 62 kDa MMP-2 mature enzyme. Where indicated, AG3340, SB-3CT or EGCG were added to the cells for 18 h. (Middle panel) Western blotting of CD44. IS-CD8^+^ cells were surface-biotinylated and were then either allowed to adhere, in serum-free medium, to plastic coated with type I collagen/gelatin (adherent, A) or remained in suspension (non-adherent, NA). Where indicated, AG3340, SB-3CT or EGCG were added to the cells. Cell lysate and medium samples were captured with streptavidin-agarose beads. CD44 was analyzed in the captured sample aliquots (50 mg total protein each) by western blotting with an antibody to the CD44 ectodomain. (Bottom panel) MMP-2 is inhibited by low concentrations of SB-3CT. α1-Antitrypsin was incubated with MMP-2. The digest samples were analyzed by reducing SDS-gel electrophoresis. Where indicated, SB-3CT was added to the samples. AG3340, 3(S)-2,2-dimethyl-4[4-pyridin-4-yloxy-benzenesulfonyl]-thiomorpholine-3-carboxylic acid hydroxamate; MT1-MMP, membrane type-1 matrix metalloproteinase; MMP-2, matrix metalloproteinase-2; SB-3CT, 2-(4-phenoxyphenylsulfonylmethyl)thiirane; EGCG, epigallocatechin-3-gallate.

To demonstrate MT1-MMP proteolysis of T cell CD44, IS-CD8^+^ T cells were surface biotinylated with membrane-impermeable biotin. The labeled cells were then allowed to either adhere to a gelatin-coated plastic or were kept in solution. The cells were then lysed and biotin-labeled CD44 was captured from the cell lysate and medium samples using streptavidine-agarose beads. The captured samples were examined by western blotting with the CD44 antibody to measure the level of the released, soluble CD44 ectodomain and the residual, membrane-anchored, cellular CD44 in the medium and the cell lysates, respectively. In addition, media samples were analyzed by gelatin zymography to determine the activation status of MMP-2. The soluble MMP-2 proenzyme is known to be directly activated by cellular MT1-MMP (21,23). To inhibit cellular MT1-MMP and, as a result, to repress the conversion of the MMP-2 proenzyme into the enzyme, the IS-CD8^+^ T cells, where indicated, were supplemented with AG3340, SB-3CT or EGCG (Fig. 1).

Consistent with previous observations (6,9), endogenous MT1-MMP was latent in non-adherent T cells, while the adhesion of T cells induced the activation of MT1-MMP. MT1-MMP activation resulted in the subsequent activation of exogenous MMP-2 and the cleavage of T cell CD44. By contrast, non-adherent T cells did not activate MMP-2 and shed cell CD44 inefficiently. AG3340 fully blocked the activation of MMP-2 and shedding of CD44 in adherent IS-CD8^+^ T cells. SB-3CT (an inefficient inhibitor of MT1-MMP) had no significant effect on either MMP-2 activation or CD44 shedding, while only an exceedingly high (500 mM) concentration of EGCG demonstrated a partial inhibition of MMP-2 activation without any significant effect on CD44 proteolysis.

SB-3CT was highly potent at inhibiting MMP-2 proteolysis of α1-antitrypsin (a sensitive and readily available protein substrate of MMPs) (24–26). In the absence of SB-3CT, MMP-2 proteolysis led to conversion of the 61 kDa α1-antitrypsin serpin into the 55 kDa degradation fragment that represented the N-terminal portion of the α1-antitrypsin molecule. In turn, a nanomolar range of concentrations of SB-3CT totally blocked the cleavage of α1-antitrypsin *in vitro* (Fig. 1).

### AG3340 inhibits the intra-islet homing of IS-CD8^+^ cells in NOD mice

**Figure 2.**
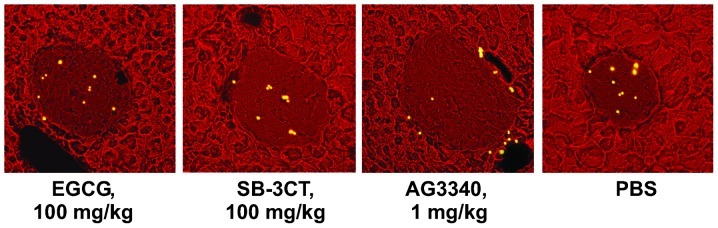
AG3340 inhibits the intra-islet homing of IS-CD8^+^ T cells. NOD mice were treated with AG3340, SB-3CT or EGCG by injection. In 30 min, this injection was followed by the injection of DiI-labeled IS-CD8^+^ T cells. After 24 h, the cryo-sections of the pancreata were examined using a fluorescence microscope. The DiI-labeled cells were ascribed their position, either at the entrance of the islet or inside the pancreatic islets, and counted. At least 100 islets per mouse (4–5 mice/group) were examined. The islets are easily recognized by their morphological characteristics including lower fluorescence and a compact, dense, structure. Representative images of the pancreatic islets from NOD mice that received an injection of DiI-labeled cells are shown. AG3340, 3(S)-2,2-dimethyl-4[4-pyridin-4-yloxy-benzenesulfonyl]-thiomorpholine-3-carboxylic acid hydroxamate; SB-3CT, 2-(4-phenoxyphenylsulfonylmethyl)thiirane; EGCG, epigallocatechin-3-gallate; NOD, non-obese diabetic; DiI, didodecyl-tetramethylindocarbocyanine perchlorate.

**Figure 3.**
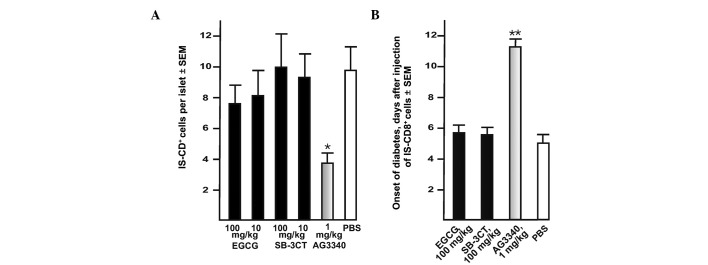
AG3340 inhibits transendothelial migration of IS-CD8^+^ T cells and delays the onset of transferred diabetes in NOD mice. (A) AG3340 inhibits the transmigration of IS-CD8^+^ cells into the pancreatic islets. Mice received AG3340, SB-3CT, EGCG or PBS 30 min prior to the injection of the cells. IS-CD8^+^ cells were labeled with DiI and then injected in NOD mice. In 24 h, the labeled cells with their intra-islet location were counted in the cryostat sections of the entire pancreas. (B) AG3340 delays the onset of adoptively transferred diabetes in NOD mice. IS-CD8^+^ cells were injected in NOD mice. Mice received AG3340, SB-3CT,EGCG or PBS by one injection every other day until they developed diabetes (approximately 1–2 weeks). The onset of diabetes was monitored daily by measuring urine glucose levels with Diastix reagent strips. Mice with urine glucose levels of ≥300 mg/dl for 3 consecutive days were considered diabetic. ^*^P=0.02, ^**^P=0.015 by Fisher’s test. AG3340, 3(S)-2,2-dimethyl-4[4-pyridin-4-yloxy-benzenesulfonyl]-thiomorpholine-3-carboxylic acid hydroxamate; NOD, non-obese diabetic; SB-3CT, 2-(4-phenoxyphenylsulfonylmethyl)thiirane; EGCG, epigallocatechin-3-gallate; DiI, didodecyl-tetramethylindocarbocyanine perchlorate.

To determine the anti-diabetic potential of the SB-3CT and EGCG non-MT1-MMP inhibitors relative to that of AG3340, NOD mice received an IP injection of the indicated concentrations of SB-3CT, EGCG or AG3340. DiI-labeled IS-CD8^+^ cells were then injected IV into the NOD mice. After 24 h, labeled IS-CD8^+^ cells were counted at the periphery and inside the islets (Fig. 2). In the absence of AG3340, T cells efficiently transmigrated into the islets. By contrast, in the presence of AG3340 T cells were detected at the islet entrance. A few cells were found inside the islets. SB-3CT and EGCG, which were used at a much higher concentration than AG3340, did not affect the homing of IS-CD8^+^ cells into the pancreatic islet (Fig. 3).

### MT1-MMP inhibitor delays development of transferred diabetes in NOD mice

To corroborate the results further, IS-CD8^+^ cells were injected in NOD mice. Prior to the cell injection (30 min), the mice received either the inhibitors or PBS (control) IP. The inhibitor injections continued every other day until the mice developed diabetes. AG3340 at a concentration as low as 1 mg/kg delayed the onset of diabetes approximately 2-fold compared with the control (Fig. 3). By contrast, there was no delay of the transferred diabetes onset in mice which received SB-3CT and EGCG, which are potent inhibitors of MMPs other than MT1-MMP.

As has been shown previously in the context of a type 2 diabetes rat model, MMP-2, MMP-12 and MT1-MMP are upregulated in diabetic males and high-fat-fed female Zucker diabetic fatty rats as compared with their non-diabetic lean counterparts (27). PD166793 [(S)-2-(4′-bromo-biphenyl-4-sulfonylamino)-3-methyl butyric acid; a broad-range inhibitor with EC_50_ values of 6100, 47, 12, 7200, 7900, 8 and 240 nM against MMP-1, MMP-2, MMP-3, MMP-7, MMP-9, MMP-13 and MT1-MMP, respectively] (28,29) preserved β cell mass, presumably, by affecting the turnover of certain extracellular matrix molecules in the islets. Despite the fact that the mechanisms of the protective effects and relative importance of the individual targets of the MMP inhibitors in T1D and in type 2 diabetes are not completely understood, it is clear that in a transfer diabetes model in NOD mice only AG3340, the antagonist of MT1-MMP, delivered clinically relevant effects. Due to the wide-range specificity of the MMP inhibitors, only a simultaneous assessment of AG3340, SB-3CT and EGCG permitted us to conclude that T cell MT1-MMP is predominant in T1D. Based on these data, it is likely that the combined effect of the individual MMPs, including MMP-2 and MMP-9, which are distinct from MT1-MMP and efficiently inhibited by SB-3CT, is less important. We conclude that MT1-MMP antagonists would be efficient in delaying T1D transfer into NOD mice. These results demonstrate the functional importance of the MT1-MMP-CD44 axis in mediating the efficiency of transendothelial migration and the homing of diabetogenic T cells into the pancreatic islets (30).

These current findings, particularly when combined with our prior results (6,9), provide a working hypothesis for the novel, anti-diabetic, application of the sharply focused, specific inhibitors of MT1-MMP. The data suggest that the inhibition of T cell MT1-MMP is a step forward in the design of novel and effective therapies for T1D. It is now likely that the pharmacological inhibition of MT1-MMP by specific antagonists will diminish the homing of T killer cells into the islets. Consequently, is possible that this favorable event would stimulate the regeneration of insulin-producing β cells in the islets (9), leading to a more positive outcome for T1D patients (31–33).

## Abbreviations:

AG3340: 3(S)-2,2-dimethyl-4[4-pyridin-4-yloxybenzenesulfonyl]-thiomorpholine-3-carboxylic acid hydroxamate
DiI: didodecyl-tetramethylindocarbocyanine perchlorate
EGCG: epigallocatechin-3-gallate
MMP-2: matrix metalloproteinase-2
MMP-9: matrix metalloproteinase-9
MT1-MMP: membrane type-1 matrix metalloproteinase
NOD: non-obese diabetic
SB-3CT: -(4-phenoxyphenylsulfonylmethyl)thiirane
T1D: type 1 diabetes
